# Discovery–dominance trade-off among widespread invasive ant species

**DOI:** 10.1002/ece3.1542

**Published:** 2015-06-17

**Authors:** Cleo Bertelsmeier, Amaury Avril, Olivier Blight, Hervé Jourdan, Franck Courchamp

**Affiliations:** 1 Ecologie, Systématique & Evolution, UMR CNRS 8079, Univ. Paris SudOrsay Cedex 91405, France; 2 Department of Ecology and Evolution, Biophore, UNIL-Sorge, University of Lausanne1015, Lausanne, Switzerland; 3 Estación Biológica de Doñana, Consejo Superior de Investigaciones Científicas41092, Sevilla, Spain; 4 Institut Méditerranéen de Biodiversité et d’Écologie marine et continentale (IMBE), Aix-Marseille Université, UMR CNRS IRD Avignon Université, UMR 237 IRD, Centre IRD NouméaBP A5, 98848, Nouméa Cedex, New Caledonia

**Keywords:** Behavioral dominance, biological invasions, discovery–dominance trade-off, exploitation, exploration, invasive ants

## Abstract

Ants are among the most problematic invasive species. They displace numerous native species, alter ecosystem processes, and can have negative impacts on agriculture and human health. In part, their success might stem from a departure from the discovery–dominance trade-off that can promote co-existence in native ant communities, *that is,* invasive ants are thought to be at the same time behaviorally dominant and faster discoverers of resources, compared to native species. However, it has not yet been tested whether similar asymmetries in behavioral dominance, exploration, and recruitment abilities also exist among invasive species. Here, we establish a dominance hierarchy among four of the most problematic invasive ants (*Linepithema humile*, *Lasius neglectus*, *Wasmannia auropunctata*, *Pheidole megacephala*) that may be able to arrive and establish in the same areas in the future. To assess behavioral dominance, we used confrontation experiments, testing the aggressiveness in individual and group interactions between all species pairs. In addition, to compare discovery efficiency, we tested the species’ capacity to locate a food resource in a maze, and the capacity to recruit nestmates to exploit a food resource. The four species differed greatly in their capacity to discover resources and to recruit nestmates and to dominate the other species. Our results are consistent with a discovery–dominance trade-off. The species that showed the highest level of interspecific aggressiveness and dominance during dyadic interactions.

## Introduction

Worldwide, ant invasions are a major threat to biodiversity (Holway et al. 2002; Lach and Hooper-Bui 2010; Rabitsch 2011; Wittman 2014). Mostly of tropical and subtropical origin, invasive ants have succeeded in colonizing every continent on Earth except Antarctica and very diverse types of habitats (Suarez et al. 2010). Native ants only rarely succeed in coexisting with invasive ants and are often displaced or can even go locally extinct (Holway et al. 2002; Lach and Hooper-Bui 2010; Rabitsch 2011; Wittman 2014). In invaded areas, the abundance of native ants can be reduced by over 90% (Porter and Savignano 1990).

So far, research has largely concentrated on describing the impacts of invasive ants on biodiversity within communities (Holway et al. 2002; Lach and Hooper-Bui 2010) and the alteration of the co-occurrence pattern of surviving species at a larger scale (Gotelli and Arnett 2000). It remains, however, unclear in many cases how invasive ants achieve this ecological dominance. A suite of research papers based on laboratory or field experiments has revealed differences between native and invasive ants in diet, aggressiveness, thermal preferences, and periods of activity (reviewed in Holway et al. 2002). However, rarely has the causal link between a certain trait difference and the displacement of native ant species been tested. High levels of aggressiveness and high interference abilities may allow invasive species to defend and monopolize resources (Holway et al. 2002). In addition, preference for sugary substances can lead to a diet higher in carbohydrates, which has been linked to higher colony growth rates and increased aggressiveness (Grover et al. 2007; Gaigher et al. 2011). All of these traits have been suggested to help invasive ants to either dominate resources or to more efficiently discover and exploit them. But behavioral dominance alone cannot explain the displacement of native ants because native ant communities can be strongly structured by competition and contain dominant species with aggressive behavior and large colony sizes (Savolainen and Vepsäläinen 1988; Andersen 1992; Parr et al. 2005; Cerdá et al. 2013). In native communities, the interaction between traits promoting interference or exploitative competition can in fact promote coexistence (Adler et al. 2007). There is a well-studied trade-off between the capacity to discover resources and the capacity to defend them (Fellers 1987; Lebrun and Feener 2007; Parr and Gibb 2012; Cerdá et al. 2013). Invasive ants may not simply excel at one or the other, but at both. The Argentine ant, *Linepithema humile*, has been shown to break this discovery–dominance trade-off in a native ant community in the western United States (Holway 1999), *that is,* the species is better at both discovering and dominating the resources than the native ant species. The violation of this discovery–dominance trade-off (Fellers 1987; Lebrun and Feener 2007; Parr and Gibb 2012; Cerdá et al. 2013) can lead to clear advantages over the species excelling in one characteristic at the systematic loss of the other. This competitive advantage over local ant species provides a direct mechanism of invasiveness (Human and Gordon 1996). However, it is unknown if all invasive species are equally good explorers and nestmate recruiters or if they differ in their relative competitive abilities related to discovering and dominating resources. If such asymmetries exist among invasive species, excelling each at different dominance components, it is likely that different invasive ant species do not invade using the same behavioral strategies. Given the high number of invasive ants, 19 are currently listed by the IUCN (IUCN ISSC Invasive Species Specialist Group, 2012), and their high impacts on biodiversity, agriculture, health, and economy (Holway et al. 2002; Lach and Hooper-Bui 2010; Rabitsch 2011), it is urgent to gain a better understanding of the mechanisms of invasiveness.

The objective of our study was to test (1) whether four of the worst invasive ants differ in their exploration and exploitation behavior, that is, their ability to discover resources and successfully recruit nestmates, (2) whether differences are related to their capacity to dominate in interference competition, (3) whether there is a discovery–dominance trade-off among invasive ants.

As a model system, we use four highly invasive ants, among a pool of several invasive ant species that have been shown to have potentially overlapping suitable areas and are likely to encounter each other in the future (Bertelsmeier et al. 2015a). They were also selected because they have been previously shown to interact aggressively and to form a linear dominance hierarchy in interference competition (Bertelsmeier et al. 2015b): *Wasmannia auropunctata *> *Lasius neglectus *> *Linepithema humile *> *Pheidole megacephala*. This made them ideal candidates to test the existence of asymmetries in different types of competitive abilities and a potential trade-off among them.

Here, we use two experiments to test the species’ capacities to discover and exploit resources. The first is an exploitation experiment, where food resources were placed directly in front of the nest to test which species is the fastest discoverer and recruiter of nestmates. The second experiment tests the species’ abilities to explore quickly their environment by separating the nest and a food source by a maze.

## Materials and Methods

### Colony collection and maintenance of laboratory colonies

The ants were collected between March and December 2012 in New Caledonia (*W. auropunctata* and *P. megacephala*) and in southern France (*L. humile* and *L. neglectus*). Experiments were conducted in December 2012 and January 2013 (for details see Table S1). Colony fragments were maintained in large plastic nest containers (55 × 35 × 25 cm) filled with substrate from the original nesting site (soil, wood, leafs) and contained several tubes of water. These boxes were kept at 24 ± 2°C with the appropriate soil moisture. The ants were fed daily with a variant of the Bhatkar diet (Dussutour and Simpson 2008).

### Exploitation experiment

**Figure 1:**
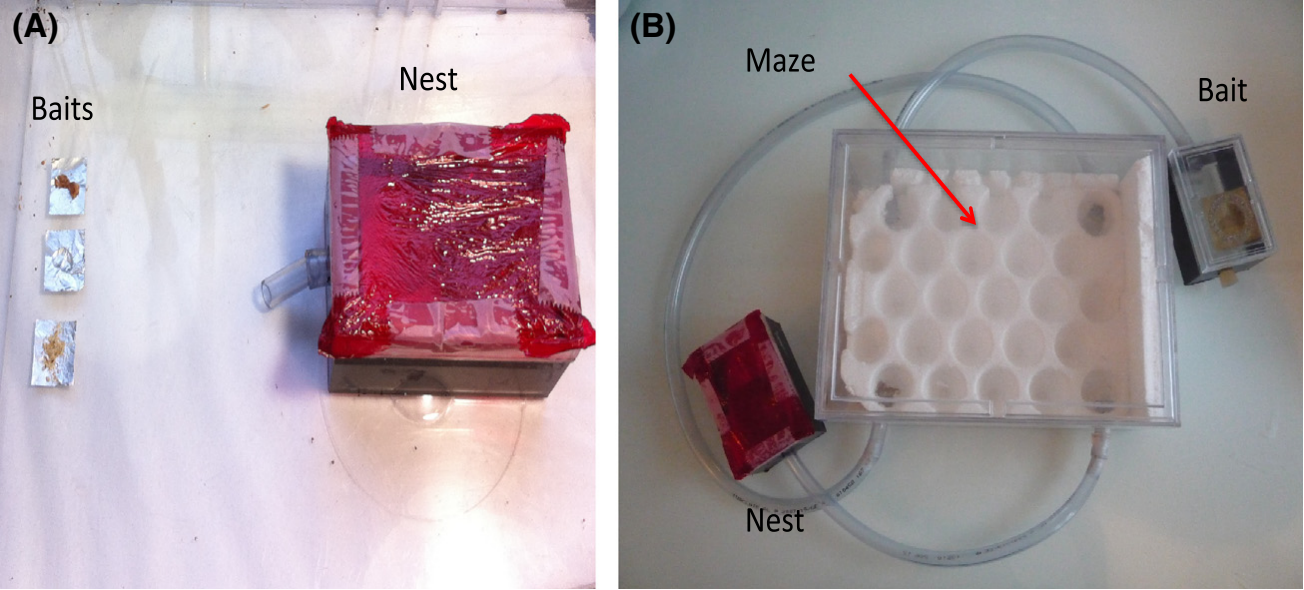
(A) Setup of the experiment testing exploitative abilities, (B) setup of the maze testing explorative abilities.

Prior to the experiment, the ants were starved for 1 week. Subsequently, 300 workers and one queen were collected and the experimental colony was placed into a small plastic nest (11 × 8 × 4.5 cm), filled with plaster at an appropriate humidity and covered with a red filter. The entrance of the nest was a small plastic tube (diameter 0.8 cm, length 3 cm), touching the foraging arena (Fig.1A). The entrance of the nest was blocked with a piece of cotton, and the ants were allowed to acclimatize for 24 h, before the cotton was removed. Three baits (tuna, sugary water, and a seed mixture) were placed on aluminum foil at a distance of approximately 6 cm from the nest entrance, in a random order. We recorded foraging behavior for 2 h with a camera taking a picture every 10 sec (i.e., 720 photos per trial). We recorded the time until discovery of the baits (arrival of the first ant), time until recruitment (defined as five workers present simultaneously at a bait), the maximum number of workers observed simultaneously at a bait, and the total number of workers observed at a bait. After each experiment, the foraging arena was cleaned and pheromone traces were eliminated with alcohol. Each experimental colony was only used in one single trial. We carried out ten replicates per species.

### Exploration experiment

**Figure 2:**
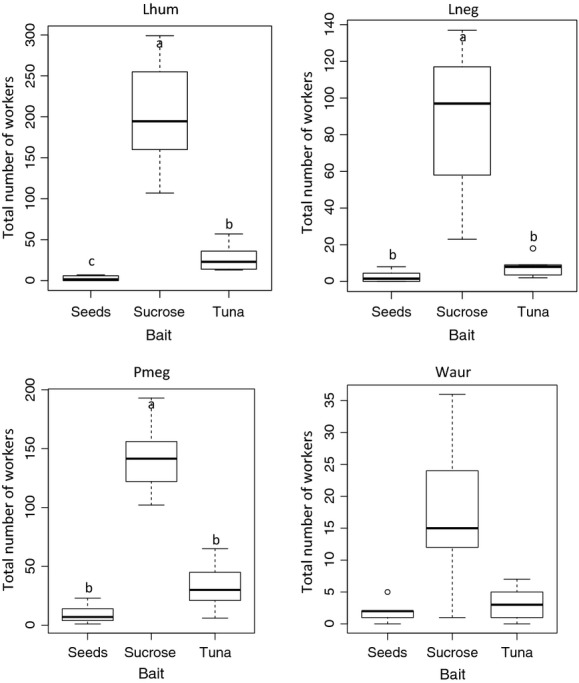
Cumulated number of workers observed during 2 h at each of three baits, averaged over the ten replicates ± SD. Lhum, *L. humile*; Lneg, *L. neglectus*; Pmeg, *P. megacephala*; Waur, *W. auropunctata*.

Prior to the experiment, experimental colonies were prepared in the same way as for the exploitation experiment (see above). The entrance of the nest was connected through a long plastic tube (diameter 0.8 cm, length 40 cm) to an exploration arena (24 × 17.5 × 10 cm) (Fig.1B). The exploration arena contained a polystyrene structure (25 holes 3 cm deep, with a diameter of 2.8 cm), which constituted a 3D “maze” that the ants needed to explore in order to find the foraging arena. At the diagonal opposite to the nest, another plastic tube (diameter 0.8 cm, length 40 cm) was connected to a small foraging arena (11 × 8 × 4.5 cm), where a bait (honeyed water) was placed. The bait was chosen because of its attractiveness to all of the four invasive ant species (exploitation experiment, Fig.2). The two other diagonals of the exploration arena were connected to each other through a longer plastic tube (diameter 0.8 cm, length 60 cm). The entrance of the nest was blocked with a piece of cotton, and the ants were allowed to acclimatize for 24 h, before the cotton was removed and the exploration arena was accessible. We recorded foraging behavior in the foraging arena for 40 h with a camera taking a picture every 2 min (in total 1200 photos per trial). We recorded the time until discovery of the baits (arrival of the first ant), time until recruitment (defined as five workers present simultaneously at a bait), and the maximum number of workers. It was not possible to record the total number of workers that had visited the bait in this experiment because the time lapse of 2 min between pictures does not allow to track individuals. After each experiment, the whole setup was cleaned and pheromone traces were eliminated with alcohol.

Each experimental colony was only used in one single trial. We carried out five replicates per species. In both experiments, colonies of *P. megacephala* contained 10% of major workers, (following Kirschenbaum and Grace 2008).

The competing colonies consisted of equal numbers of individuals because the individual is the basic unit of the collective organization of the colony and seemed therefore appropriate when testing recruitment capacities. In addition, previous research has failed to demonstrate a link between body size and recruitment speed (Spacek Godoy and Marques de Camargos 2013).

### Interference competition

To test the relationship between discovery/exploitation and behavioral dominance, we used data from a previous study on interference competition among invasive ants (Bertelsmeier et al. 2015b). The study used classical dyadic confrontation experiments in Petri dishes, using single worker and group (10 workers) interactions. Based on the confrontations of each species with each of six other invasive ants, a “survival” index (SI) was calculated for each species, reflecting its capacity to survive all pairwise interactions. In addition, a “killing” index (KI) was calculated, reflecting the species’ capacity to kill its opponents in pairwise interactions. As, both SI and KI were initially based on pairwise interactions of seven species, we recalculated both indices for only the four species in our study. This was achieved by taking into account only the scores of each species achieved when confronted with the three other species used in our study.

### Analyses

In cases where “discovery” or “recruitment” was not observed over the duration of the experiment, the maximum time (40 h) was assigned to the colony. As we used rank-based nonparametric tests, this attributes the last discovery rank to all colonies (equal score) that did not discover the resource in the given time (or failed to recruit to it).

Prior to statistical analysis of the differences in SI and KI among species, we examined all data distributions using the Shapiro–Wilk W-test for normality. Because the residuals did not conform to a normal distribution, we used the nonparametric Kruskal–Wallis rank sum test, adjusting for multiple comparisons with a Kruskal multiple comparison test of the kruskalmc() function included in the pgirmess package in R, v. 2.15.

Subsequently, we tested if the species’ capacity to discover resources and/or recruit to them are correlated to their behavioral dominance score, using a linear regression. As both dominance indices, SI and KI, are very tightly correlated (*r*^2^ = 0.9738), we only used one behavioral dominance score, SI. The discovery/recruitment variables are measured in time, and therefore, higher values indicate slower, less competitive species.

For the sake of presentation and interpretation, we preferred here to present these variables so that a higher score would equal a higher ability, in order to be able to compare with dominance scores (which are higher for more dominant species). We therefore transformed the variables into a discovery or recruitment score (total time of the experiment minus time until event [either discovery or recruitment]). In case of a very fast discovery, the score approaches the maximum time of the experiment, and when the event did not occur, the score equals 0.

## Results

### Exploitation experiment

In the exploitation experiment, the highest number of workers was observed for all four species at the sugar bait (Fig.2). The differences in diet preferences for sugar were significant for all species, except *W. auropunctata* (Kruskal–Wallis test: *L. humile χ*^2^_(2)_ = 25.8525, *P *<* *0.0001, *L. neglectus χ*^2^_(2)_ = 18.0008, *P *=* *0.0001234, *P. megacephala χ*^2^_(2)_ = 23.3969, *P *<* *0.0001, *W. auropunctata χ*^2^_(2)_ = 5.1038, *P *=* *0.07793). Pairwise comparisons among baits revealed a further preference of *L. humile* for tuna over seeds (Fig.2), but in the two remaining species, *L. neglectus* and *P. megacephala*, the difference between tuna and seeds was not significant. Given that all four species were mostly attracted by sucrose and always discovered it first, in the following section, the variable “time to discovery” always refers to the time until the first worker arrived on the sucrose bait. The cumulative number of workers at baits is the total number of ants that visited baits (all three baits pooled).

**Figure 3:**
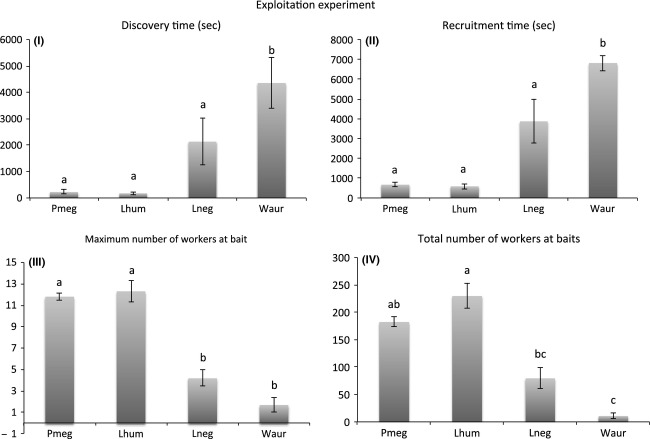
Differences among species in (I) time until discovery of the bait, (II) time until recruitment with five workers simultaneously at a bait, (III) the maximum number of workers observed simultaneously on baits, (IV) the total, cumulated number of workers visiting baits over 2 h. Different letters denote significant pairwise comparisons in the post hoc multiple comparison Kruskal–Wallis test. Values are given ± s.e.m.

Species differed significantly in discovery time (*χ*^2^_(3)_ = 22.347, *P *<* *0.0001) and recruitment time (*χ*^2^_(3)_ = 30.188, *P *<* *0.0001), in both cases *L. humile* and *P. megacephala* were again the fastest, followed by *L. neglectus* and *W. auropunctata,* although not all pairwise comparisons were significant (Fig.3). Further, species differed significantly in the maximum number of workers observed simultaneously at baits (Kruskal test *χ*^2^_(3)_ = 30.694, *P *<* *0.0001), with *P. megacephala* and *L. humile* recruiting the highest number of workers (Fig.3). In addition, species differed significantly in the total, cumulated number of workers that were recruited over the 2 h of the experiment (*χ*^2^_(3)_ = 30.188, *P *<* *0.0001), with again *P. megacephala* and *L. humile* recruiting the highest number of workers (Fig.3).

### Exploration experiment

**Figure 4:**
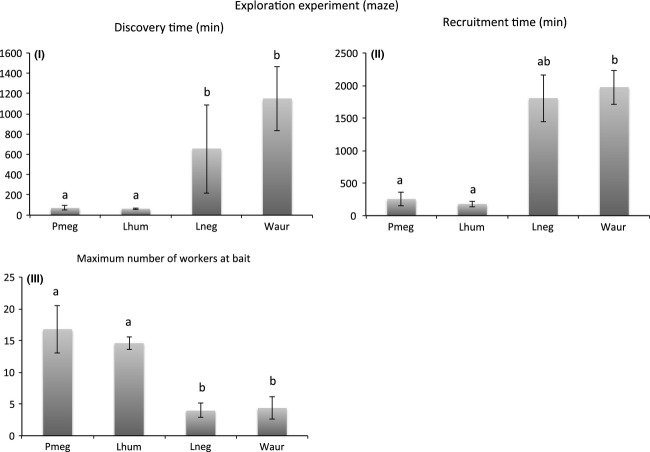
Differences among species in (I) time until discovery of the bait, (II) time until recruitment with five workers simultaneously at a bait, (III) the maximum number of workers visiting simultaneously baits. Different letters denote significant pairwise comparisons in the post hoc multiple comparison Kruskal–Wallis test. Values are given ± SEM.

Species differed significantly in discovery time (*χ*^2^_(3)_ = 14.819, *P *=* *0.002) and recruitment time (*χ*^2^_(3)_ = 14.672, *P *=* *0.002), in both cases *L. humile* and *P. megacephala* were again the two fastest, followed by *L. neglectus* and *W. auropunctata* together (Fig.4). Further, species differed significantly in the maximum number of workers observed simultaneously at baits (Kruskal test *χ*^2^_(3)_ = 13.531, *P *=* *0.004), with here again *P. megacephala* and *L. humile* recruiting the highest number of workers simultaneously over the 40 h of observation (Fig.4).

### Discovery–dominance trade-off

**Figure 5:**
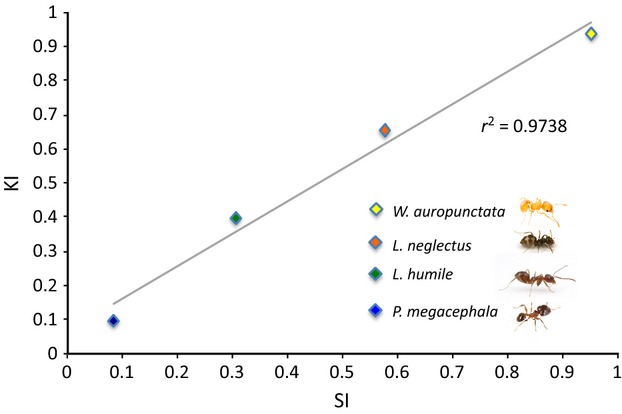
Dominance hierarchy in interference competition. SI, survival index; KI, killing index.

The four species form a linear dominance hierarchy, with SI and KI being tightly correlated (Fig.5).

**Figure 6:**
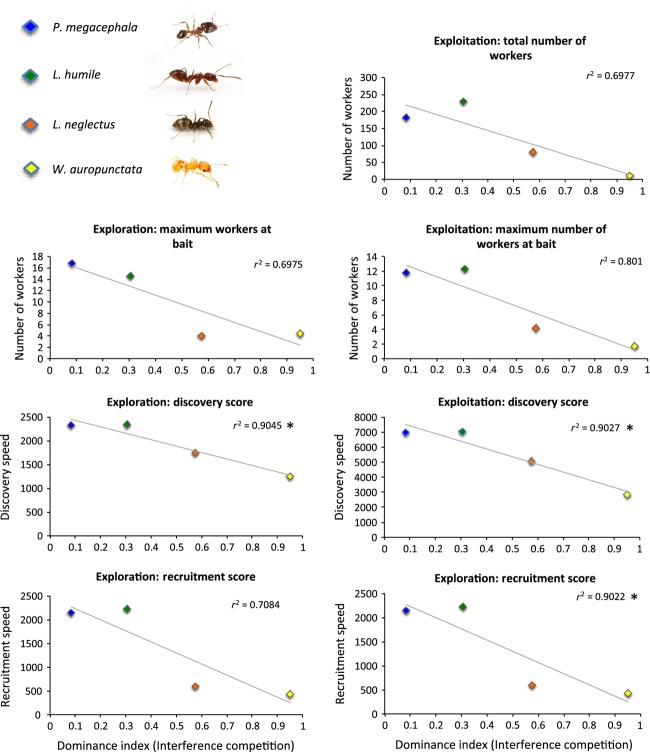
Dominance in interference competition versus exploration or exploitation abilities. Significant correlations are marked with a star (*).

Discovery and exploitation abilities were negatively related to dominance (Fig.6). In the exploitation experiment, the most dominant species had a lower discovery (*r*^2^ = 0.927, *P *=* *0.033) and recruitment speed (*r*^2^ = 0.922, *P *=* *0.033). They also showed a tendency to recruit a lower maximum number of workers simultaneously, but these correlations were not significant (*r*^2^ = 0.801, *P *=* *0.061) and a lower total number of workers (*r*^2^ = 0.698, *P *=* *0.106).

The same pattern was observed in the 40 h exploration experiment, where discovery and exploitation abilities were negatively related to dominance (Fig.6). The most dominant species had a lower discovery score (*r*^2^ = 0.905, *P *=* *0.032). However, the tendency for lower recruitment speed (*r*^2^ = 0.708, *P *=* *0.103) and recruitment a lower number of workers simultaneously were not significant (*r*^2^ = 0.698, *P *=* *0.165).

## Discussion

### Main findings

We observed asymmetries in exploitation and recruitment among major invasive species. The four invasive ant species, *W. auropunctata*, *L. neglectus*, *L. humile,* and *P. megacephala*, share many life-history traits and are all widespread invasive species capable of displacing many native species (Holway et al. 2002; Lach and Hooper-Bui 2010; Rabitsch 2011). Yet, they differ greatly in their capacity to discover resources and their capacity to exploit them by quickly recruiting a high number of nestmates are correlated. Combining these results with the hierarchy based on interference competition, we were able to show a negative relationship between both exploitation and exploration on the one hand and the capacity to dominate behaviorally on the other hand. This supported the idea of a negative discovery–dominance relationship among four of the worst invasive ant species. Consistently, *W. auropunctata* has been shown to be the most dominant species in dyadic confrontation experiments (Bertelsmeier et al. 2015b), surviving almost all interactions and killing most opponents. In the present study, this species was by far the slowest exploring and the least recruiting species. In contrast, *L. neglectus* and *L. humile*, both of which had lower dominance scores in dyadic confrontations, had higher exploration and discovery abilities. Last, *P. megacephala* had the lowest dominance score but was the fastest exploring and the best exploiting species of the four.

Our results thus suggest that, even though invasive ants may break discovery–dominance trade-offs in native communities (Holway 1999), there is a discovery–dominance trade-off among invasive ants. In uninvaded communities, the existence of a discovery–dominance trade-off is thought to promote species coexistence (Adler et al. 2007), in spite of otherwise similar ecological niches. Therefore, the results of our study may be interpreted as a possibility of co-existence among the four highly invasive ants, studied here. Recent studies have suggested that these four invasive species could find suitable climatic conditions in the same regions of the world, arguing for potential interactions between them in multiply invaded zones (Bertelsmeier and Courchamp 2014; Bertelsmeier et al. 2015a). However, the current distribution of highly invasive ants seems exclusive so far, at least at a local scale (LeBrun et al. 2007; Krushelnycky and Gillespie 2010; Spicer Rice and Silverman 2013). A possibility is that no ultimate “top invasive” ant species exists. Different species could have superior capacities related to exploitation or to interference. Local patches may be dominated by one species or the other, depending on environmental factors not considered in our study. This is consistent with the observation that *P. megacephala* and *W. auropunctata* form a mutually exclusive mosaic distribution in New Caledonia, each dominating different areas (Chazeau et al. 2000; Le Breton 2003). This pattern would not be predicted by the behavioral dominance hierarchy alone, where *W. auropunctata* is the top dominant species and should exclude *P. megacephala* from all the invaded area.

### Limitations and future directions

Several limitations are inherent to the laboratory-based experimental approach used here. For example, the discovery–dominance trade-off might depend on habitat complexity (Sarty et al. 2006). In addition, resource size and distribution can be important in determining the outcome of competition (Gibb 2005; LeBrun 2005; Parr and Gibb 2010). The neutral laboratory setup does also not offer the possibility to study potentially mitigating biotic interactions, such as competitors or parasites that are known to influence the dominance–discovery trade-off (Porter et al. 1997; Lebrun and Feener 2007). Similarly, differences in temperature preferences may alter dominance relationships in the field (Cerda et al. 1997; Lessard et al. 2009; Arnan et al. 2012). For example, *L. humile* is superior at interference competition in the laboratory, but is nonetheless displaced by *P. chinensis* in areas where the climatic suitability is low for *L. humile* and high for *P. chinensis* (Spicer Rice and Silverman 2013). The results are contingent on the environmental conditions, while laboratory temperature, humidity, and diet are not necessarily representative of natural conditions (a limitation shared by many laboratory-based studies). It would be very interesting if future studies could carry out field-based tests of this trade-off. Yet, it will not be possible to construct a hierarchy among those four species simultaneously in the field. Yet, perhaps future research could investigate pairwise interactions at contact zones to confirm the existence of the trade-off under natural conditions. In addition, future experimental work could explore the effect of asymmetrical population densities in competition.

Here, we compared the two sets of traits (interference competition vs. exploitation competition) among invasive species. Although these invasive species demonstrate a clear trade-off among them, it is conceivable that they all break that trade-off relative to native ants by being above average in their ability for both sets of traits. A way to test this interesting hypothesis, although logistically very challenging, would be to compare interference and exploitation abilities of all four species to the native species of their respective invaded communities. Also, our results on the discovery–dominance trade-off among invasive ants are based on interactions among only four species and it would be interesting to consider a potential trade-off among a greater group of invasive ants in order to investigate the generality of this pattern.

In addition, whether this trade-off exists in the native ranges of these invasive species is very intriguing and, if so, where do these invasive species lie along the trade-off curve ought to be assessed. How these trade-off curves vary across local communities remains unknown. In the future, if enough data from a variety of natural communities become available, it might be possible to develop a theoretical framework, comparing these trade-off curves and improving predictions of ant invasions in particular communities.

### Implications

Generally, invasive ants are perceived as a group of species sharing similar life-history traits, such as polygyny, omnivory, and unicoloniality (Holway et al. 2002). Their aggressive behavior is often invoked when explaining the displacement of native species (Passera 1994; Human 1999; McGlynn 1999; Holway et al. 2002; Cremer et al. 2008). Frequently, invasive ants are compared to native ants but rarely among each other. However, this study shows that they do not only differ in the abilities at interference competition but also in their abilities at exploitative competition. Although it has become accepted that one possible mechanism explaining invasiveness is the violation of the dominance–dominance trade-off (Parr and Gibb 2010), it is quite unexpected to find this trade-off among invasive ants.

An interesting question is whether invasive ants generally violate the trade-off in the invaded habitat or whether the mechanism of invasiveness differs among species, some superior at exploitation/discovery, others at interference competition, and others again at both. So far, it has been suggested that *Anoplolepis gracilipes* may break the trade-off in certain types of habitat (Sarty et al. 2006), and similarly, *Solenopsis invicta* has been shown to break the trade-off sometimes, but not always (Feener et al. 2008), relative to ant species in the invaded habitat. Although interference and exploitative competitions have been recognized as the two pillars of ant community ecology (Cerdá et al. 2013), few studies have investigated exploitation and exploration, because interference is much easier to detect and interference competition between two species is a somewhat more direct interaction (Parr and Gibb 2010).

To conclude, our results have shown a discovery–dominance trade-off among four highly invasive ants. This opens new research question regarding the mechanisms of invasiveness in ants. In particular, it shows that invasive ants may employ different strategies to become invasive, some relying more on their interference superiority, some others on exploitation ascendency. In addition, the interaction of different invasive ant species deserves further attention, especially in light of ongoing global changes and increasing species introductions (Simberloff et al. 2013). It is likely that areas suitable to several invasive ants will suffer from multiple invasions in the future, and it is crucial to further improve the understanding of interactions among different invasive species in order to better manage these invasions.

## Supporting Information

**Table S1.** Ant collection for the experiments.
