# Supplementary material for: Discovery–dominance trade-off among widespread invasive ant species
Source: Ecol Evol. 2015 Jun 17;5(13):2673–83. doi: 10.1002/ece3.1542 (PMC4523362; doi:10.1002/ece3.1542)
Supplement: Supplementary file 1 [file ece30005-2673-sd1.docx]

| \| **Collection date** \| \| --- \| \| 24-26/03/2012 \| \| 24-26/03/2012 \| \| 17/05/2012 \| \| 20/03/2012 \| \| 02/07/2012 \| \| 18/05/2012 \| \| 21/03/2012 \| | \| **Collector** \| \| --- \| \| Olivier Blighht \| \| Olivier Blighht \| \| Hervé Jourdan \| \| Hervé Jourdan \| \| Hervé Jourdan \| \| Hervé Jourdan \| \| Hervé Jourdan \| | \| **Species** \| \| --- \| \| *Lasius neglectus* \| \| *Linepithema humile* \| \| *Pheidole megacephala* \| \| *Pheidole megacephala* \| \| *Wasmannia auropunctata* \| \| *Wasmannia auropunctata* \| \| *Wasmannia auropunctata* \| | \| **Number of nests*** \| \| --- \| \| 6 \| \| 10 \| \| 4 \| \| 3 \| \| 2 \| \| 2 \| \| 2 \| | \| **Locality** \| \| --- \| \| Allauch, France \| \| Ceyreste, France \| \| Parc Forestier, Nouméa, New Caledonia \| \| Anse Vata, Nouméa, New Caledonia \| \| Ouen Toro, Nouméa, New Caledonia \| \| Parc Forestier, Nouméa, New Caledonia \| \| Ouen Toro, Nouméa, New Caledonia \| | \| **GPS coordinates** \| \| --- \| \| 43º22'18.50"N ; 5º30'44.95"E \| \| 43º12'39.45"N//5º37'53.71"E \| \| 22°15'31.19"S//166°27'28.19"E \| \| 22°18'2.62"S//166°26'39.88"E \| \| 22°18'18.66"S//166°26'56.33"E \| \| 22°15'35.35"S//166°27'32.75"E \| \| 22°18'18.66"S//166°26'56.33"E \| |
| --- | --- | --- | --- | --- | --- | --- | --- | --- | --- | --- | --- | --- | --- | --- | --- | --- | --- | --- | --- | --- | --- | --- | --- | --- | --- | --- | --- | --- | --- | --- | --- | --- | --- | --- | --- | --- | --- | --- | --- | --- | --- | --- | --- | --- | --- | --- | --- | --- | --- | --- | --- | --- | --- |

| *^*^* In unicolonial species, individuals can mix freely between nests. |
| --- |
| We defined as 'nests', colonies that were more than 10m apart. |
